# Carbonized Apples and Quinces Stillage for Electromagnetic Shielding

**DOI:** 10.3390/nano14231882

**Published:** 2024-11-23

**Authors:** Mila Milenkovic, Warda Saeed, Muhammad Yasir, Dusan Milivojevic, Ali Azmy, Kamal E. S. Nassar, Zois Syrgiannis, Ioannis Spanopoulos, Danica Bajuk-Bogdanovic, Snežana Maletić, Djurdja Kerkez, Tanja Barudžija, Svetlana Jovanović

**Affiliations:** 1 Vinča Institute of Nuclear Sciences—National Institute of the Republic of Serbia, University of Belgrade, P.O. Box 522, 11000 Belgrade, Serbia; mila.milenkovic@vin.bg.ac.rs (M.M.);; 2 Department of Computing Science, Microrobotics and Control Engineering, Carl von Ossietzky Universität Oldenburg, 26129 Oldenburg, Germany; warda.saeed@uni-oldenburg.de; 3 Department of Chemistry, University of South Florida, Tampa, FL 33620, USAspanopoulos@usf.edu (I.S.); 4 Faculty of Physical Chemistry, University of Belgrade, Studentski Trg 12–16, 11158 Belgrade, Serbia; 5 Department of Chemistry, Biochemistry and Environmental Protection, Faculty of Science, University of Novi Sad, Trg Dositeja Obradovića 3, 21000 Novi Sad, Serbia

**Keywords:** graphene, graphene oxide, biowaste, carbonization, electromagnetic interference shielding

## Abstract

Electromagnetic waves (EMWs) have become an integral part of our daily lives, but they are causing a new form of environmental pollution, manifesting as electromagnetic interference (EMI) and radio frequency signal leakage. As a result, the demand for innovative, eco-friendly materials capable of blocking EMWs has escalated in the past decade, underscoring the significance of our research. In the realm of modern science, the creation of new materials must consider the starting materials, production costs, energy usage, and the potential for air, water, and soil pollution. Herein, we utilized biowaste materials generated during the distillation of fruit schnapps. The biowaste from apple and quince schnapps distillation was used as starting material, mixed with KOH, and carbonized at 850 °C, in a nitrogen atmosphere. The structure of samples was investigated using various techniques (infrared, Raman, energy-dispersive X-ray, X-ray photoelectron spectroscopies, thermogravimetric analysis, BET surface area analyzer). Encouragingly, these materials demonstrated the ability to block EMWs within a frequency range of 8 to 12 GHz. Shielding efficiency was measured using waveguide adapters connected to ports (1 and 2) of the vector network analyzer using radio-frequency coaxial cables. At a frequency of 10 GHz, carbonized biowaste blocks 78.5% of the incident electromagnetic wave.

## 1. Introduction

Aside from the traditional forms of pollution caused by chemicals in gaseous, liquid, or solid states, such as micro- and nano-plastics, modern society is now grappling with a new type of pollution, i.e., electromagnetic waves (EWs). The proliferation of devices in our daily lives, the expansion of telecommunication infrastructure, and the evolution of wireless technologies has underscored the critical need for innovative materials capable of blocking the propagation of EWs. Electromagnetic interference (EMI) is particularly detrimental in the case of sensitive measurement devices, potentially leading to a reduction in the instrument’s lifespan, unwanted interference signals, and prolonged measuring times. 

Thus, new materials, in the form of powder, foil, and fabric, which can efficiently block electromagnetic wave propagation, have become more critical in recent years [1,2,3]. Studies have suggested that metals, metal composites, and conductive polymers have excellent EMI shielding properties [4,5,6]. New nanomaterials, such as silk-flower-like NiO with a hierarchical structure, showed a reflection loss (R_L_) of –65.1 dB at 13.9 GHz due to its hierarchical and porous structures, multiple scattering effects from nanosheets, folds and voids, different impedance matching characteristics, and clustered defects, where oxygen vacancies could disrupt the charge distribution balance and induce dipole polarization and related relaxations [7]. A flexible, semiconductive device was fabricated using carbon nanotubes with a tunable electromagnetic wave absorption frequency [8]. However, these materials are often sensitive to environmental conditions, such as humidity [9], or have limited processability, e.g., conductive polymer [10]. Furthermore, the question of the sustainability of the production, carbon footprint, and recyclability of these new materials is open. 

Graphene and its derivates show promising EMI shielding properties [11,12,13]. Defect-free single-layer graphene shows an EMI shielding efficiency (SE) of 2.27 dB [14], while few-layered graphene, graphene doped with more electronegative atoms, and multilayer film made of small and large graphene flakes show the ability to block 99% of the incident electromagnetic wave [11,15,16,17,18]. The main shielding mechanism of graphene is absorption [14], which is a desirable shielding mechanism compared to materials that reflect EMWs, such as metals, which cause secondary pollution [19]. Apart from electrical conductivity, both high dielectric loss and low density make graphene a very favorable material as an EMW absorber, while a derivate of graphene, graphene oxide (GO), possesses oxygen functional groups that induce strong dipolar polarization and interfacial polarization, improving microwave attenuation at GHz frequencies [20]. Composites based on graphene and its derivates block EMWs by absorption, reflection, and multiple reflections [21,22]. 

Combining graphene or graphene oxide with a metal-based nanostructure such as silver nanowires (AgNWs) enhances the conductivity and improves EMI SE, from 17 dB as measured for reduced graphene oxide to 38 dB as obtained for a composite of graphene with AgNWs [23]. By adding AgNWs as a middle layer between two reduced graphene oxide layers, Kumar et al. created a sandwich structure, with an electrical conductivity of 6.5 × 10^4^ S m^−1^, able to block EMWs dominantly by absorption of waves [23]. Considering that metals are efficient shielding materials based mainly on EMW reflection, conducive graphene-based composites also show desirable EMI SE, with the advantage that the shielding mechanism often involves both reflection and absorption [22]. 

Herein, we investigated the possibility of fruit biowaste usage as a starting material in producing new, environment-friendly EMI shielding materials. Therefore, we selected a side product from the fruit schnapps manufacturing process. These byproducts are called distillery stillage. Stillage is discharged in large quantities by distilleries: for 1 L of produced alcohol, between 8 and 15 L of stillage is generated [24]. Due to high levels of compounds with nitrogen and various organic molecules, these waste materials must be properly managed, which demands additional costs in the disposal of or treatment of stillage [25,26]. One solution for stillage management is to produce fertilizer or livestock feed, both low-value products [27]. Recently developed technology for recovering phenolic acids has been reposted, contributing to more efficient stillage valorization [28].

In this study, we used stillage collected after distillation of apple and quince schnapps and pyrolyzed at 850 °C. We analyzed the structural and morphological properties, as well as the ability of the materials to block EMWs in the X-band frequency region. To investigate the structure of carbonized materials at both qualitative and quantitative levels, several spectroscopic techniques were used: infrared, Raman, energy-dispersive X-ray, X-ray photoelectron spectroscopies, thermogravimetric analysis, and BET surface area analyzer. It was previously reported that biochar produced from lignin increased the SE of cement [29], while sewage sludge biochar showed an SE value >10 dB [29,30]. By exploring the possibility of converting stillage into value-added, new, and highly demanded material, this study aims to answer the need for these new sustainable products and give a new perspective on reducing the environmental burden of stillage discharge.

## 2. Materials and Methods

Samples of carbonized biomass were produced starting from residual waste material after fruit schnapps distillation, i.e., distillery stillage. Two different fruit schnapps residual distillery stillages were used. Namely, residuals from apples and quinces were collected. The distillery “Skrbic”, Nestin, Republic of Serbia, donated the stillages. 

The experimental procedure was conducted in the following stages:

1. Stillages were first diluted, using demineralized water produced by the Millipore Milli-Q^®^ water system, Burlington, MA, USA. The volume ratio of stillage to water was 1:2.
2. Diluted stillage was mechanically homogenized: the mixtures were homogenized mechanically using a kitchen chopper with a power of 2000 W in 4 cycles for 15 min. Large particles and fragments of the starting materials that could lead to inhomogeneity in the resulting material were removed by filtration through filter paper.
3. Homogenized, diluted stillage was filtrated: the resulting solution was filtered through a Whatman Filter Paper, Grade 4 (20–24 µm particle retention, Whatman plc, Maidstone, UK). The collected filtrate was used for further production, while the material left on the surface of the filter paper was discarded.
4. Drying of the filtrates: after collecting filtrates, they were dried at atmospheric pressure at 85 °C. Consequently, black powders were obtained.
5. Pre-carbonization step: to achieve carbonization, dried biomass was mixed with KOH (reagent grade, 90%, flakes, Sigma Aldrich, St. Louis, MO, USA) in a mass ratio of 1:1.
6. Carbonization step: samples were heated for 1 h at 850 °C in the stream of N_2_.
7. Cleaning step: after carbonization, the samples were washed with demineralized water to remove KOH. Specifically, black powders obtained after carbonization were immersed in water. The powders were separated from water by simple decantation after clean water was added to the powder. The procedure was repeated until the pH was 7.
8. After KOH was removed, black powders were collected and dried at 70 °C at a reduced pressure.

The sample produced from residuals that remained after ethanol distillation in the process of apple schnapps production was named BA, and the other sample produced from quince schnapps residuals was named BQ. These powders were then used for further analysis.

Raman spectra were recorded on a DXR Raman microscope (Thermo Fisher Scientific, Waltham, MA, USA). Each spectrum was obtained at room temperature using a 532 nm excitation line with a power of 2 mW. The data were analyzed through a spectrometer equipped with 800 lines/mm aperture and a 50 μm pinhole. The acquisition time was 10 × 10 s.

For Fourier-transform infrared spectroscopy (FTIR) analysis, powdered biomass samples were mixed with KBr and compressed into pellets. FTIR spectra were recorded using an FTIR Spectrometer (Thermo Scientific Nicolet iS20 FTIR Spectrometer, Thermo Scientific™, Waltham, MA, USA), in the range of 4000–400 cm^−1^ at 32 scans per spectrum and a resolution of 4 cm^−1^.

The thermal stability of the BA and BQ samples was investigated using a Mettler Toledo TGA/DSC 1 instrument (Mettler Toledo, Columbus, OH, USA). Per each analysis, around 3 mg of powder samples were used. Samples were heated from 40 °C to 750 °C at a heating rate of 5 K min^−1^ at a nitrogen flow rate of 20 mL min^−1^. Each measurement was repeated two times. 

The structural analysis of the synthesized material was conducted using the X-ray diffraction (XRD) method by employing a SmartLab^®^ X-ray diffractometer (Rigaku Co., Tokyo, Japan), with Cu Kα radiation at a continuous scanning mode (40 kV, 30 mA, and λ = 1.542 Å). Measurements were made in the various diffraction angles ranging from 6 to 35° with a step size of 0.02° and a measurement speed of 2° min^−1^.

Scanning electron microscopy with an energy-dispersive X-ray spectroscopy (SEM–EDS) was performed on an INCAx-act LN2-free analytical silicon drift detector of characteristic X-rays with the PentaFET^®^ Precision and Aztec 4.3 software package (Oxford Instruments, Oxford, UK) connected to a TESCAN Mira3 XMU (20 kV, SE detector, Brno, Czech Republic). Samples of pyrolyzed biomass were deposited on double-sided conductive and adhesive type and were analyzed. 

To investigate the structure of BA and BQ in detail, X-ray Photoelectron Spectroscopy (XPS) was used. These measurements were performed using a Physical Electronics Industries PHI 5400 LS XPS (Physical Electronics, Chanhassen, MN, USA) equipped with a 10-360 Spherical Capacitor Analyzer (SCA). The SCA was set at aperture 2 small, yielding an analysis area of 600 microns. Aluminum K-alpha X-rays were used as a source. The power was set to 350 watts. SCA pass energy was set to 178.95 eV. The eV/step was 0.250 eV. Powdered BA and BQ samples were pressed onto Indium foil, which served as support. 

The specific surface area was obtained by performing nitrogen (N_2_) adsorption. The mesopore and micropore volumes were determined using the BJH and *t*-test methods, and utilizinga Quantachrome autosorb TMiQ (Quantachrome Instruments, Boynton Beach, FL, USA) surface area analyzer.

**Figure 1:**
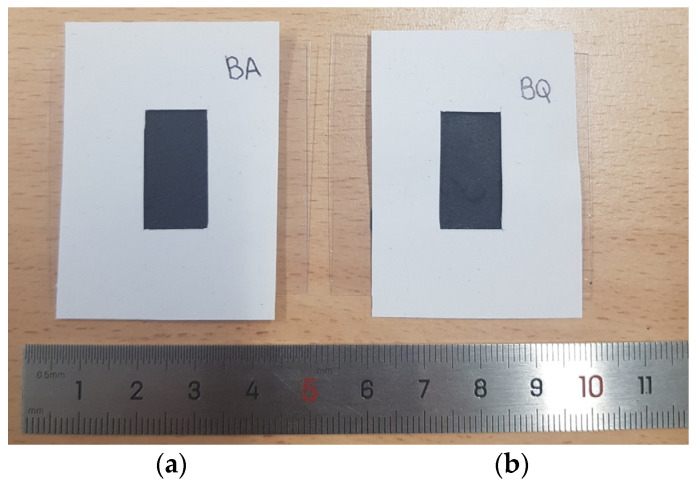
Prepared films of BA (**a**) and BQ (**b**) samples.

To investigate the shielding efficiency, BA and BQ powders were weighed and mixed with a sodium silicate resin to produce a homogenous paste. The concentration of prepared BA and BQ pastes was 0.4 g mL^−1^ (0.004 g/10 µL). BA and BQ pastes were deposited on a 0.2 mm thick plexiglass sheet covered with a paper mold and formed into a 22.86 mm × 10.16 mm thin film. The thickness of the films was also 0.2 mm, corresponding to the thickness of the plexiglass sheet. This size corresponds to the inner dimensions of WR-90 waveguide adapters which were used for the electromagnetic shielding measurement. The prepared films of BA and BQ samples are shown in Figure 1. The transmission coefficients in the X-band (8–12 GHz frequency range) were measured using a Rohde & Schwarz ZVA 24 Vector Network Analyzer (VNA, Munich, Germany).

The measurement setup is shown in Figure S1. It consisted of two WR90 waveguide adapters connected to ports 1 and 2 of the VNA using RF coaxial cables. The thin film formed was placed between the two waveguide adapters and the S_21_ scattering parameter was measured for both films, as previously reported [31]. 

The shielding effectiveness due to transmission (SE_T_), Shielding Effectiveness due to dissipation (SE_A_) and Shielding Effectiveness due to reflection (SE_R_) are plotted using Equations (1)–(3):

SE_T_ = −S_21_ dB (1)

SE_R_ = −10log(1 − |S_11_|^2^) (2)

SE_A_ = −10log(|S_21_|^2^/(1 − |S_11_|^2^)) (3)

where S_11_ and S_21_ represent the reflection and transmission coefficients, respectively, which have been obtained from the Vector Network Analyzer after the measurement of the biomass samples. 

## 3. Results and Discussion 

**Figure 2:**
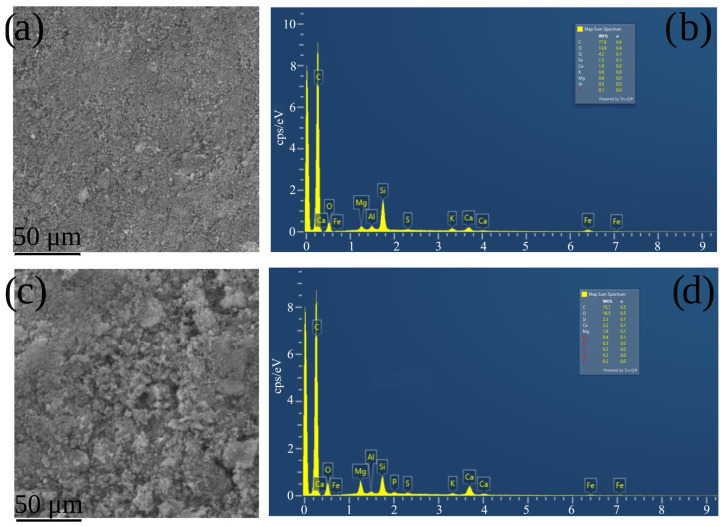
SEM images and associated EDS spectra of BA (**a**,**b**) and BQ (**c**,**d**), respectively.

To investigate the chemical composition, SEM–EDS analysis was initially performed. Figure 2 shows SEM images of the pyrolyzed biomass samples. It can be observed that both BA and BQ are homogeneous and have granular morphology.

According to EDS analysis (Figure 2b,d and Table 1), the main chemical elements in both samples are C (77.82 to 75.66 wt%) and O (13.90 and 16.49 wt%), while other elements, such as Mg, Ca, Si, S, K, and Al, were also detected but in very small amounts. The presence of toxic metals in biochar provides important information for estimating the environmental impact of these newly produced materials [32]. EDS analysis shows that Fe is present in 1.3 wt% in BA, while other heavy metals were not detected. 

**Figure 3:**
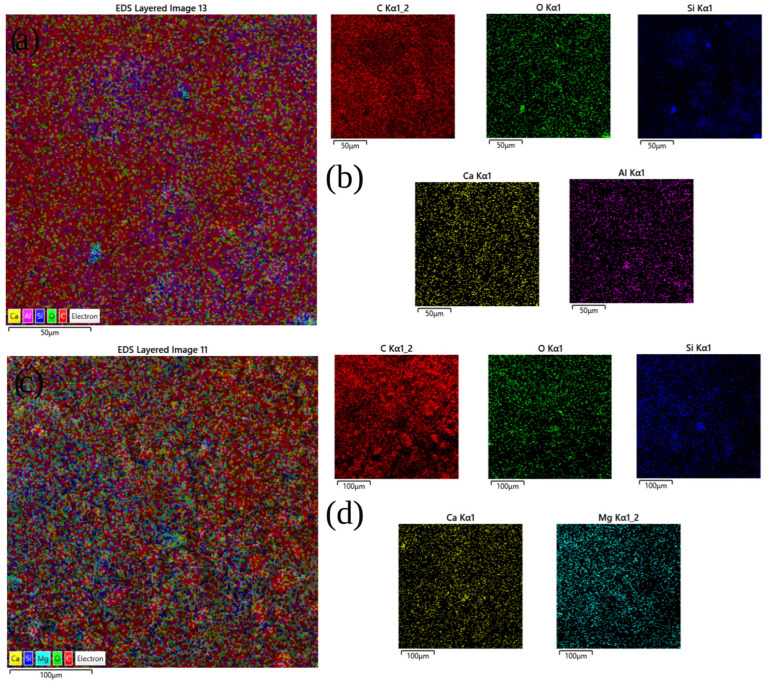
SEM–EDS maps of BA (**a**,**b**) and BQ (**c**,**d**) for C, O, Si, Ca, and Mg.

EDS maps were obtained to investigate homogeneity in terms of chemical composition (Figure 3). These results indicated an equal distribution of all detected elements on the surface of both samples. 

**Figure 4:**
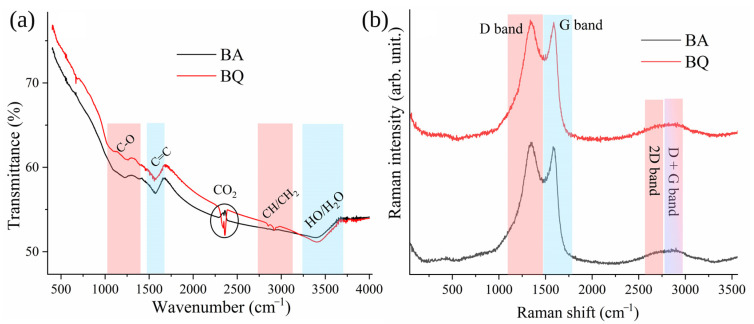
FTIR (**a**) and Raman (**b**) spectra of BA (black curve) and BQ (red curve).

FTIR spectroscopy was used to investigate the structure of carbonized biomass samples, and collected spectra are presented in Figure 4. All spectra show the band at 3412 cm^−1^. This band is assigned to OH groups, attached to hydroxyl groups of the carbon skeleton of the material, or could be associated with physically absorbed water. Two low-intensity bands at 2922 and 2851 cm^−1^ are also observed. These bands are commonly observed in FTIR spectra of various carbon-based nanomaterials, in graphene oxide, graphene quantum dots, and carbon nanotubes [33,34], and stem from the asymmetric and symmetric stretching vibrations of alkyl and aliphatic CH_2_ bonds [35]. These bands are prominent in the FTIR spectrum of BQ but could hardly be observed in the BA spectrum. This change is associated with a lower content of methyl groups in the sample of BA [36]. All spectra show a prominent band at 1570 cm^−1^ resulting from vibrations of C=C bonds and proves the presence of sp^2^ domains in all samples. A low-intensity band at 1384 cm^−1^ is detected and assigned to C-O bonds in COOH groups. Additionally, two bands at 1240 and 1110 cm^−1^ are associated with stretching vibrations of C-O bonds in epoxy and alkoxy groups, respectively [33,34]. 

Raman spectroscopy was used to investigate the structure of BA and BQ samples, and spectra are presented in Figure 4b. The two most prominent bands are observed in all spectra, one at 1349 cm^−1^ and the second at 1585 cm^−1^. The first band stems from defects in graphitic, sp^2^ structure due to grain edges, various vacancies, amorphous carbon, and functional groups that create sp^3^ sites [37,38,39]. This band is called the D-band and its intensity is associated with the level of structural disorder. The second band is called the graphitic or G-band. This band stems from the first-order scattering of the E_2g_ phonon of the bonds between sp^2^ carbon atoms [40]. The band around 2700 cm^−1^ was also detected. This band is called G’ band or 2D which involves a two-phonon double resonance Raman scattering process [41]. An additional low-intensity band could be noticed at 2875 cm^−1^. This band is named the D + G band and is observed in other graphene samples produced from biomass [42]. Applying the Knight and White equation [43], crystalline size (La) was calculated and its values are presented in Table 2. These calculations show that an adequate size of the basal plane is similar for BA and BQ samples obtained from different starting materials and comparable to GO produced using the modified Hummers method. 

Figure 4a,b indicates that BA and BQ possess both sp^2^ and sp^3^ C in their structure, as well as various oxygen-containing functional groups, such as hydroxyl, carbonyl, and carboxyl. 

**Figure 5:**
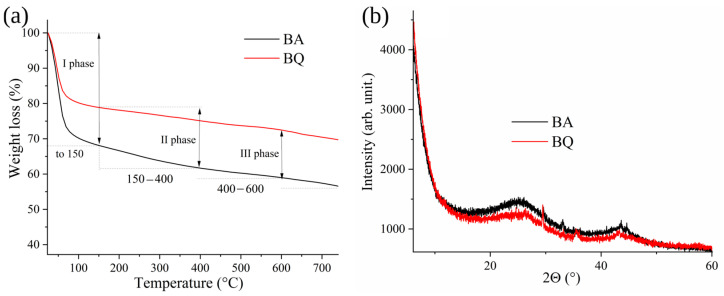
TGA curves (**a**) and XRD patterns (**b**) of BA (black) and BQ (red).

Figure 5a shows the TGA curves of carbonized biomass samples. Both TGA curves are similar to previously observed thermograms obtained for carbonized rice husks [45]. The pyrolysis process is divided into three main phases: dehydration, devolatilization, and carbonization [46,47]. In the first stage, up to 150 °C, a major weight loss of 21.08% for BQ and 32.01% for BA was measured. This weight loss is associated with the evaporation of physically absorbed water, including inbound water [48]. Additionally, degradation of volatile compounds with a small molecular weight could also occur at this stage [49,50]. In the second step, in the temperature range of 150–400 °C, the minor mass weights were measured: 3.80% for BQ and 6.27% for BA. In graphene-based materials, weight loss in this temperature range is often associated with the decomposition of oxygen-containing functional groups, such as OH and epoxy groups [51]. These weight losses could be associated with the decomposition of oxygen-containing functional groups, such as OH, and epoxy groups from the graphitic regions of carbonized biomass [52,53,54]. As a third step (in the temperature range of 400–600 °C), weight losses of 2.64% for BQ, and 2.73% for BA were measured. FTIR analysis showed the presence of different oxygen-containing functional groups, such as carboxyl, carbonyl, and hydroxyl. It is assumed that these groups were decomposed during stages 2 and 3. Thus, the BQ sample showed less, while BA showed significantly larger amounts of the total % of oxygen-containing groups. At the final stage, at 740 °C, the residual sample weight was 69.73% for BQ and 56.12% for BA. These results indicate that the BQ sample is terminally the most stable with the lowest content of functional groups. 

**Figure 6:**
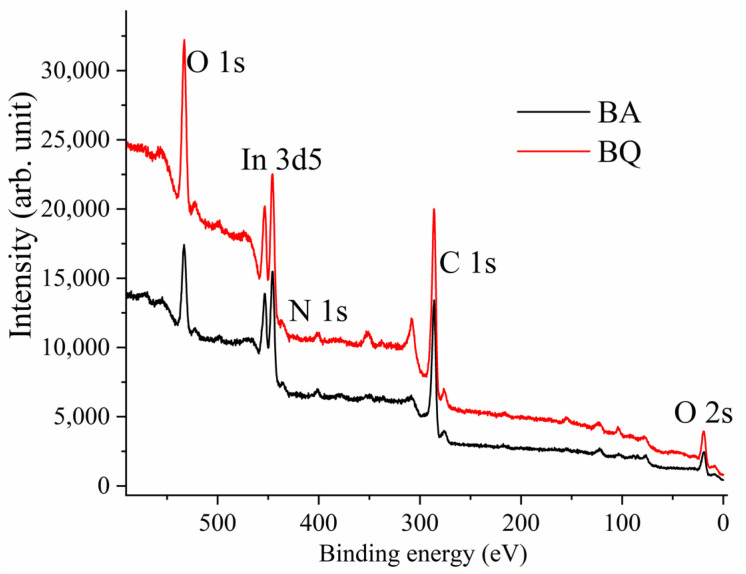
Comparison of XPS survey spectra for BA and BQ.

**Figure 7:**
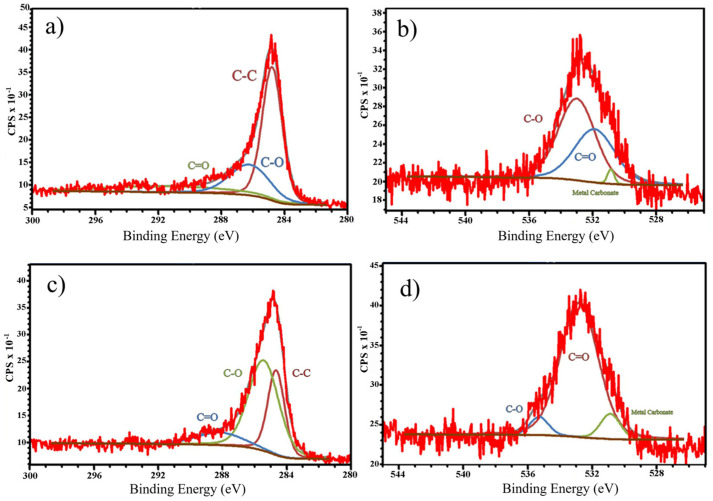
High-resolution spectra of C 1s, and O 1s regions for BA (**a**,**b**) and BQ (**c**,**d**) samples.

XRD patterns of BA and BQ show two diffraction peaks in the 2θ range of 25.5° and 43.5° (Figure 5b). These bands correspond to different types of crystalline graphite: (002) and (100) planes, respectively [55,56]. Notably, the (002) lattice diffraction peak patterns are more pronounced. These peaks suggest the formation of the graphitic crystalline structure during the carbonization process [57,58]. The higher intensity of the peaks at 25.5° for BA than in the BQ sample is related to a higher-degree graphitic crystalline structure. Both samples show a peak at 29.46° assigned to the (104) plane of CaCO_3_ [59]. The experimental data were compared with the standard ICDD database (#00-005-0586). Element Ca was observed in EDS spectra of BQ and BA samples, but higher at and mass% was detected in BQ (Table 1), analogous to the intensity of the peak at 29.46° in the XRD pattern. The structure of carbonized biomasses was investigated using XPS and the results of these measurements are displayed in Figure 6 and Figure 7. Figure 6 shows survey spectra and reveals that both samples contain the following elements: C, O, N, and In. The indium (In) presence in both spectra is due to the In foil being used as a sample support. The regions indicated by C1s, O1s, and N1s were further analyzed (see Figure 7). 

In both spectra, in the C 1s region, three main contributions were identified (Figure 7a,d): carbon in C-C/C=C bonds at 284.8 eV and 284.6 eV, the C−O carbon attribute to both epoxy and hydroxy groups at 286.2 and 285.4 eV, and the C=O carbon at 291.1 and 288.2 eV, in BA and BQ spectra, respectively. Bonds identified in the C 1s region were confirmed by inspection of the O 1s region, where oxygen in single and double bonds were identified in the BA spectrum at 533.0 for C−O, and 531.9 at C=O eV, C-O, ≅ 535.3 eV, and C=O, ≅532.8 eV in BQ. In C-O/C=O bonds, O was also detected in carbonates in both samples. 

The regions indicated by C1s, O1s, and N1s were further analyzed (see Figure 7 and Figure S2, Supplementary Materials). In both spectra, in the C 1s region, three main contributions were identified (Figure 7a,c): carbon in C-C/C=C bonds, at 284.8 eV and 284.6 eV, the C−O carbon attribute to both epoxy and hydroxy groups, at 286.2 and 285.4 eV, and the C=O carbon at 291.1 and 288.2 eV, in BA and BQ spectra, respectively. Bonds identified in the C 1s region were confirmed by inspection of the O 1s region (Figure 7b,d), where oxygen in single and double bonds were identified in the BA spectrum at 533.0 for C−O, and at 531.9 eV for C=O, C-O, ≅535.3 eV, and C=O, ≅532.8 eV in BQ. In C-O/C=O bonds, O was also detected in carbonates in both samples.

The relative abundances of identified elements and bonds are reported in Table 3. 

**Figure 8:**
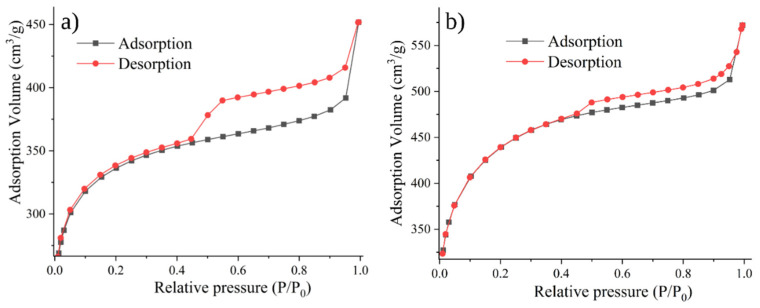
Nitrogen adsorption/desorption isotherms of BA (**a**) and BQ (**b**).

Gas sorption properties are presented in Table 4, while N_2_ adsorption/desorption isotherms are shown in Figure 8. 

For BA and BQ, N_2_ isotherms exhibit a type IV isotherm with an H4 hysteresis loop according to the IUPAC classification [60,61]. According to Table 4, the BQ sample possesses a larger specific surface area and total pore volume than BA, while the average pore volumes are similar. According to results fromthe *t*-test and pore size distribution calculations, both materials are considered as mesoporous adsorbents, according to the IUPAC classification. Measured specific surface area (SSA) (BET) values are significantly larger compared to those obtained for oak wood and oak bark biochar (1–3 m^2^ g^−1^), as well as to commercial activated carbon (1000 m^2^ g^−1^) [62] or those obtained from empty fruit bunch biochar of oil palm (2.57 m^2^ g^−1^) [63], plumeria rubra (1581 m^2^ g^−1^) [64], while higher values were reported for pine nutshells (2093 m^2^ g^−1^) [65], coal tar (2887 m^2^ g^−1^) [66], spruce bark (2385 m^2^ g^−1^) [67], and molecule urea (2261 m^2^ g^−1^) [68].

**Figure 9:**
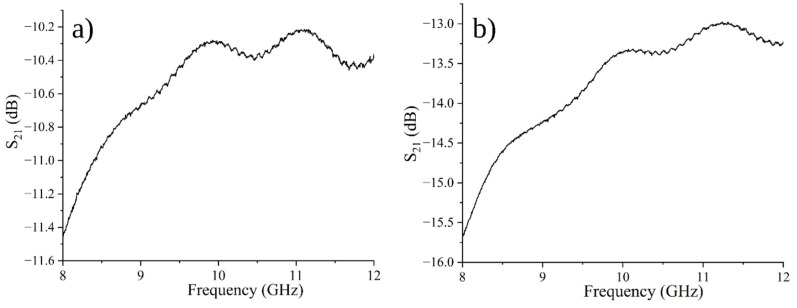
Transmission coefficient plots for BA (**a**) and BQ (**b**) samples measured in frequency range from 8 to 12 GHz.

Transmission coefficient values for BA and BQ films deposited on a Plexiglas sheet with paper mold are measured in the X-band. Subsequently, the measured transmission coefficient for the Plexiglas sheet with paper mold is subtracted from the former measurements to obtain the transmission coefficient data for BA and BQ samples only. From Figure 9a,b, it can be observed that the transmission coefficient values are higher for the BA sample than for the BQ sample. These results imply that BQ is a better shield than BA since it can block more EM waves. Electromagnetic shielding is due to the reflection, transmission and dissipation in the samples. Moreover, the transmission coefficient values increase at higher frequencies in both cases. The maximum variation in the S_21_ parameter for BA is 1.1 dB and for BQ samples its value is around 2.4 dB. These results are compliant with the findings reported in Table 3, since BQ has almost 4.5 times more at% of metal carbonate than the BA sample. This implies that BQ is more conductive than BA and hence provides higher shielding effectiveness than the BA sample. BA and BQ samples provide a shielding effectiveness of 10.7 dB and 13.5 dB, respectively, which corresponds to a blockage of 69.4% and 78.5% of the incident electromagnetic wave, respectively, at 10 GHz. 

**Figure 10:**
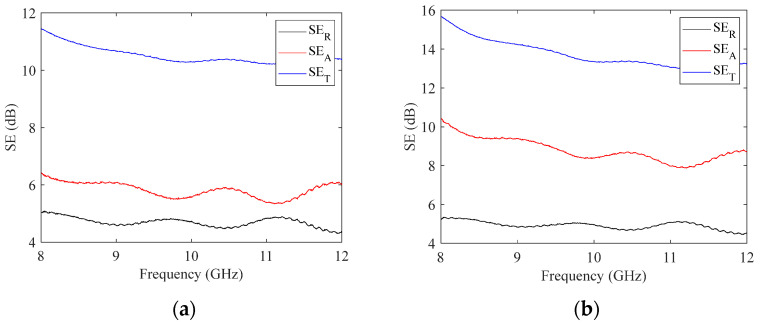
EMI performance of BA (**a**) and BQ (**b**): SE_R_, SE_A,_ and SE_T_, in the frequency range 8–12 GHz.

The total shielding effectiveness, including its components due to reflection, transmission, and dissipation, is plotted in Figure 10. 

The absorption loss for BQ is higher than for BA, while the reflective component is almost the same for both samples. Due to the more dissipative nature of the BQ sample, the shielding effectiveness values are higher for the BQ sample compared to the BA sample, hence making it a better contender for EMI shielding.

**Scheme 1:**
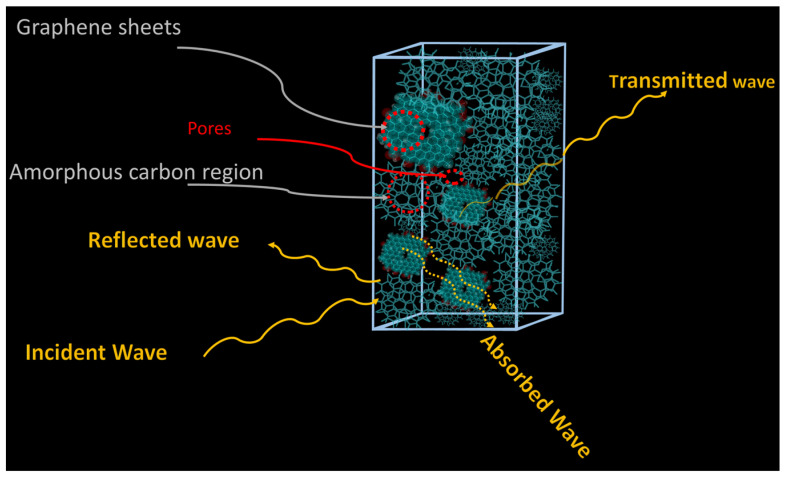
Schematic illustration of EMW shielding activity of BA or BQ samples: incident wave was partially reflected, absorbed, and transmitted.

Considering the structural analyses of BA and BQ samples, it was observed that both materials mainly contain C and O elements. Carbon atoms in both samples create sp^2^ domains and are also a part of various functional groups, such as carbonyl, carboxyl, epoxy, and alkoxy. In Scheme 1, the suggested structure of BA and BQ is presented, indicating the presence of both sp^2^ domains, oxygen-containing functional groups (red balls), and amorphous carbon. XPS analysis indicated that, in BA samples, the largest at% of C is in C-C/C=C bonds (56.43 at%), while in BQ only 29.98 at% are involved in creating the same bonds. Additionally, XRD patterns indicated higher degrees of graphitic structure in BA samples while Raman spectra support these findings. After analyzing the BET surface area, it can be seen that both samples showed large SSA values, which exceeded the values of various types of biochar reported in the literature. Amongst the samples produced, SSA for BQ is almost 30% higher than BA. In Scheme 1, porosity is also indicated. Analysis of S_21_ scattering parameters in the X-band showed that both samples are able to block the propagation of EMW. A higher shielding efficiency was measured in the BQ sample. Considering that structural analysis indicated the presence of graphene domains, and only surface area analysis showed a significant difference in the material’s properties, it is suggested that improvement in the shielding efficiency is associated with the inner, porous structure. In Scheme 1, the EMW shielding mechanism is presented in both dissipation of EMWs, marked as “absorbed wave”, and reflection. 

In summary, the existence of pores inside shielding materials improves their shielding efficiency [69,70]. When EMWs strike the interface between graphene and pores filled with air, EMWs are transmitted partially and partially reflected [70]. Those transmitted are then reflected and transmitted, while the process is repeatedly infinitely between graphene surfaces in the porous structure. With a higher free surface, and larger pore volume in BQ samples, the reflection area for incident EMWs is enlarged, making the propagation pathway of incident waves longer [70], which improves the shielding process. Yang et al. showed that carbonized biomass with graphene had a surface area of 699.1 m^2^g^−1^ and an average pore size similar to BA and BQ (2.83 nm) [71]. They conclude that the impedance matching with the free space entry of incident EMWs into materials could speed up, and pores offer channels for EMW penetration and transmission, which all together enhance multiple scattering and EMW attenuation [71]. Morphology was also recognized as one of the key factors in the EMI shielding of NiCo_2_O_4_ [72]. Shi et al. concluded that one of the most prominent factors in EMI shielding of 3D-printed carbon-based composite [73] are the porous structures, inside of which EMWs travel a longer distance, promoting EMW absorption by enhancing wave reflections and multiple reflections in the pores and leading to EMW attenuation in the form of Joule heat [74]. 

## 4. Conclusions

Materials with a large accessible surface area are produced starting from two secondary waste products from apple and quince processing. They mainly contain C and O atoms, while other elements (Al, Mg, Si, P, S, K, Ca) are also detected in less than 5 wt%. Amongst heavy metals, only Fe was detected (1.3 wt%), indicating that the produced biochar is environmentally acceptable in terms of heavy metal toxicity. C atoms are incorporated in C-C/C=C bonds and oxygen-containing functional groups. Samples BA possess a larger content of sp^2^ C atoms than BQ, while BQ possess more O-functional groups, particularly C=O. Due to the large accessible surface area, porous structure, and graphene domains, both BA and BQ samples have the ability to block up to 78.5% of incident EMWs. Absorption loss is the main shielding mechanism. The materials obtained show great promise as an ecological and sustainable solution for new products, and will block the propagation of EMWs.

## Figures and Tables

**Table 1:** Elemental composition of BA and BQ samples in wt% and atomic%.

BA			BQ		
Element	wt%	Atomic%	Element	wt%	Atomic%
C	77.82	85.18	C	75.66	83.05
O	13.90	11.43	O	16.49	13.59
Mg	0.56	0.30	Mg	1.90	1.03
Al	0.48	0.24	Al	0.25	0.12
Si	4.21	1.97	Si	2.33	1.09
S	0.11	0.04	P	0.26	0.11
K	0.62	0.21	S	0.21	0.09
Ca	0.99	0.32	K	0.28	0.09
Fe	1.30	0.31	Ca	2.22	0.73

**Table 2:** Position of G bands, I_D_/I_G_ ratios, and crystalline size (La) values for BA and BQ.

Sample	G Position	I_D_/I_G_	La = 4.4 (I_D_/I_G_)^−1^
BA	1583	0.96	4.58
BQ	1588	0.98	4.48
GO	1580	0.88–0.95 [34]	5.00
Graphite	1575	0.04 [44]	110

**Table 3:** Results of deconvolution of C 1s, O 1s, and N 1s peaks and atomic% of identified bonds in BA and BQ samples.

Sample	C1s			O1s		N1s	
Bond	C-C/C=C	C=O	C-O	C-O	Metal Carbonate	C=O	C-NH_2_
BA	56.43	17.36	26.21	57.17	1.89	40.95	100
BQ	29.98	12.68	57.34	5.77	8.47	85.76	100

**Table 4:** Gas sorption properties of BA and BQ samples.

Parameter	BQ	BA
SSA (BET) (m^2^g^−1^)	1618	1260
Micropore *t*-test (cm^3^g^−1^)	0.70	0.52
Micropore HK method (cm^3^g^−1^)	0.74	0.55
BJH Total pore volume (cm^3^g^−1^)	0.22	0.21
Average pore diameter (nm)	2.19	2.22
Total pore volume (cm^3^g^−1^) for pores <nm diameter	0.89	0.70

## Data Availability

Datasets analyzed in the current study are available in the Zenodo repository (https://doi.org/10.5281/zenodo.13884540).

## Supplementary Materials

The following supporting information can be downloaded at: https://www.mdpi.com/article/10.3390/nano14231882/s1, Figure S1: Waveguide-based Measurement Setup for the measurement of shielding of biomass samples; Figure S2. High-resolution spectra of N1s regions for BA (a) and BQ (b) samples.

## References

[1] Zhao B., Hamidinejad M., Wang S., Bai P., Che R., Zhang R., Park C.B. (2021). Advances in electromagnetic shielding properties of composite foams. J. Mater. Chem. A.

[2] Shukla V. (2019). Review of electromagnetic interference shielding materials fabricated by iron ingredients. Nanoscale Adv..

[3] Jeon Y.-J., Yun J.-H., Kang M.-S. (2022). Analysis of Electromagnetic Shielding Properties of a Material Developed Based on Silver-Coated Copper Core-Shell Spraying. Materials.

[4] Hung F. (2019). The effect of Sn-Al-C composite powders on the electromagnetic interference shielding of capsule building materials. IOP Conf. Ser. Mater. Sci. Eng..

[5] Sathish Kumar K., Rengaraj R., Venkatakrishnan G.R., Chandramohan A. (2021). Polymeric materials for electromagnetic shielding—A review. Mater. Today Proc..

[6] Ji H., Zhao R., Zhang N., Jin C., Lu X., Wang C. (2018). Lightweight and flexible electrospun polymer nanofiber/metal nanoparticle hybrid membrane for high-performance electromagnetic interference shielding. NPG Asia Mater..

[7] Liu P., Hong Ng V.M., Yao Z., Zhou J., Lei Y., Yang Z., Lv H., Kong B.L. (2017). Facile Synthesis and Hierarchical Assembly of Flowerlike NiO Structures with Enhanced Dielectric and Microwave Absorption Properties. ACS Appl. Mater. Interf..

[8] Liu J.-H., Huang Y.-S. (2023). Development of Microwave Filters with Tunable Frequency and Flexibility Using Carbon Nanotube Paper. Nanomaterials.

[9] Auffan M., Rose J., Wiesner M.R., Bottero J.-Y. (2009). Chemical stability of metallic nanoparticles: A parameter controlling their potential cellular toxicity in vitro. Environ. Pollut..

[10] Nezakati T., Seifalian A., Tan A., Seifalian A.M. (2018). Conductive Polymers: Opportunities and Challenges in Biomedical Applications. Chem. Rev..

[11] Shen B., Zhai W., Zheng W. (2014). Ultrathin Flexible Graphene Film: An Excellent Thermal Conducting Material with Efficient EMI Shielding. Adv. Funct. Mater..

[12] Jia H., Yang X., Kong Q.-Q., Xie L.-J., Guo Q.-G., Song G., Liang L.-L., Chen J.-P., Li Y., Chen C.-M. (2021). Free-standing, anti-corrosion, super flexible graphene oxide/silver nanowire thin films for ultra-wideband electromagnetic interference shielding. J. Mater. Chem. A.

[13] Oliveira F.M., Luxa J., Bouša D., Sofer Z., Gusmão R. (2022). Electromagnetic Interference Shielding by Reduced Graphene Oxide Foils. ACS Appl. Nano Mater..

[14] Hong S.K., Kim K.Y., Kim T.Y., Kim J.H., Park S.W., Kim J.H., Cho B.J. (2012). Electromagnetic interference shielding effectiveness of monolayer graphene. Nanotechnology.

[15] Zhang L., Alvarez N.T., Zhang M., Haase M., Malik R., Mast D., Shanov V. (2015). Preparation and characterization of graphene paper for electromagnetic interference shielding. Carbon.

[16] Shahzad F., Kumar P., Yu S., Lee S., Kim Y.-H., Hong S.M., Koo C.M. (2015). Sulfur-doped graphene laminates for EMI shielding applications. J. Mater. Chem. C.

[17] Lin S., Ju S., Shi G., Zhang J., He Y., Jiang D. (2019). Ultrathin nitrogen-doping graphene films for flexible and stretchable EMI shielding materials. J. Mater. Sci..

[18] Kumar P., Shahzad F., Yu S., Hong S.M., Kim Y.-H., Koo C.M. (2015). Large-area reduced graphene oxide thin film with excellent thermal conductivity and electromagnetic interference shielding effectiveness. Carbon.

[19] Jovanović S., Huskić M., Kepić D., Yasir M., Haddadi K. (2023). A review on graphene and graphene composites for application in electromagnetic shielding. Graphene 2D Mater..

[20] Hong X., Peng T., Zhu C., Wan J., Li Y. (2021). Electromagnetic shielding, resistance temperature-sensitive behavior, and decoupling of interfacial electricity for reduced graphene oxide paper. J. Alloys Compd..

[21] Shen B., Zhai W., Tao M., Ling J., Zheng W. (2017). Graphene-based sandwich structures for frequency selectable electromagnetic shielding. ACS Appl. Mater. Inter..

[22] Zhou E., Xi J., Liu Y., Xu Z., Guo Y., Peng L., Gao W., Ying J., Chen Z., Gao C. (2017). Large-area potassium-doped highly conductive graphene films for electromagnetic interference shielding. Nanoscale.

[23] Kumar P., Shahzad F., Hong S.M., Koo C.M. (2016). A flexible sandwich graphene/silver nanowires/graphene thin film for high-performance electromagnetic interference shielding. RSC Adv..

[24] Mikucka W., Zielińska M. (2020). Distillery Stillage: Characteristics, Treatment, and Valorization. Appl. Biochem. Biotechnol..

[25] Gebreeyessus G.D., Mekonnen A., Alemayehu E. (2019). A review on progresses and performances in distillery stillage management. J. Clean. Prod..

[26] Sajbrt V., Rosol M., Ditl P. (2010). A Comparison of Distillery Stillage Disposal Methods. Acta Polytech..

[27] Mikucka W., Zielińska M., Bułkowska K., Witońska I. (2022). Valorization of distillery stillage by polyphenol recovery using microwave-assisted, ultrasound-assisted and conventional extractions. J. Environ. Manag..

[28] Yasir M., di Summa D., Ruscica G., Natali Sora I., Savi P. (2020). Shielding Properties of Cement Composites Filled with Commercial Biochar. Electronics.

[29] Yasir M., Savi P. (2020). Shielding Effectiveness of Biochar Composites at Microwave Frequency. Biochar.

[30] Savi P., Yasir M. (2020). Waveguide measurements of biochar derived from sewage sludge. Electron. Lett..

[31] Li H., Lu X., Yuan D., Sun Y., Erden F., Wang F., He C. (2017). Lightweight flexible carbon nanotube/polyaniline films with outstanding EMI shielding property. J. Mater. Chem. C.

[32] Godlewska P., Ok Y.S., Oleszczuk P. (2021). THE DARK SIDE OF BLACK GOLD: Ecotoxicological aspects of biochar and biochar-amended soils. J. Hazard. Mater..

[33] Jovanovic S., Bonasera A., Dorontic S., Zmejkoski D., Milivojevic D., Janakiev T., Todorovic Markovic B. (2022). Antioxidative and Photo-Induced Effects of Different Types of N-Doped Graphene Quantum Dots. Materials.

[34] Huskić M., Kepić D., Kleut D., Mozetič M., Vesel A., Anžlovar A., Bogdanović D.B., Jovanović S. (2024). The Influence of Reaction Conditions on the Properties of Graphene Oxide. Nanomaterials.

[35] Coates J. (2006). Interpretation of Infrared Spectra, A Practical Approach. Encyclopedia of Analytical Chemistry.

[36] Jiang J., Yuan M., Xu R., Bish D.L. (2015). Mobilization of phosphate in variable-charge soils amended with biochars derived from crop straws. Soil. Till Res..

[37] Kim N.H., Kuila T., Lee J.H. (2013). Simultaneous reduction, functionalization and stitching of graphene oxide with ethylenediamine for composites application. J. Mater. Chem. A.

[38] Park O.-K., Hahm M.G., Lee S., Joh H.-I., Na S.-I., Vajtai R., Lee J.H., Ku B.-C., Ajayan P.M. (2012). In Situ Synthesis of Thermochemically Reduced Graphene Oxide Conducting Nanocomposites. Nano Lett..

[39] Cheruku R., Bhaskaram D.S., Govindaraj G. (2018). Variable range hopping and relaxation mechanism in graphene oxide sheets containing sp3 hybridization induced localization. J. Mater. Sci. Mater. Electron..

[40] Ferrari A.C., Basko D.M. (2013). Raman spectroscopy as a versatile tool for studying the properties of graphene. Nat. Nanotechnol..

[41] Park J.S., Reina A., Saito R., Kong J., Dresselhaus G., Dresselhaus M.S. (2009). G′ band Raman spectra of single, double and triple layer graphene. Carbon.

[42] Rajagopal R., Komiyama M., Borhan A. (2021). Preparation of Graphene Oxide from Lignin by Gel Combustion Method and Its Performance as Supercapacitor. E3S Web Conf..

[43] Knight D.S., White W.B. (1989). Characterization of diamond films by Raman spectroscopy. J. Mater. Res..

[44] Kim S.-G., Park O.-K., Lee J., Ku B.-C. (2013). Layer-by-layer assembled graphene oxide films and barrier properties of thermally reduced graphene oxide membranes. Carbon Lett..

[45] Yiga V.A., Nuwamanya A., Birungi A., Lubwama M., Lubwama H.N. (2023). Development of carbonized rice husks briquettes: Synergy between emissions, combustion, kinetics and thermodynamic characteristics. Energy Rep..

[46] Setter C., Oliveira T.J.P. (2022). Evaluation of the physical-mechanical and energy properties of coffee husk briquettes with kraft lignin during slow pyrolysis. Renew. Energy.

[47] Setter C., Silva F.T.M., Assis M.R., Ataíde C.H., Trugilho P.F., Oliveira T.J.P. (2020). Slow pyrolysis of coffee husk briquettes: Characterization of the solid and liquid fractions. Fuel.

[48] Ojha D.K., Kumar V.S.P., Vinu R. (2021). Analytical pyrolysis of bagasse and groundnut shell briquettes: Kinetics and pyrolysate composition studies. Bioresour. Technol. Rep..

[49] Ogwang I., Kasedde H., Nabuuma B., Kirabira J.B., Lwanyaga J.D. (2021). Characterization of Biogas Digestate for Solid Biofuel Production in Uganda. Sci. Afr..

[50] Jagnade P., Panwar N.L., Agarwal C. (2023). Experimental Investigation of Kinetic Parameters of Bamboo and Bamboo Biochar Using Thermogravimetric Analysis Under Non-isothermal Conditions. BioEnergy Res..

[51] Yap P.L., Kabiri S., Tran D.N.H., Losic D. (2019). Multifunctional Binding Chemistry on Modified Graphene Composite for Selective and Highly Efficient Adsorption of Mercury. ACS Appl. Mater. Interf..

[52] Melamane X., Strong J., Burgess J. (2007). Treatment of Wine Distillery Wastewater: A Review with Emphasis on Anaerobic Membrane Reactors. SAJEV.

[53] Farivar F., Lay Yap P., Karunagaran R.U., Losic D. (2021). Thermogravimetric Analysis (TGA) of Graphene Materials: Effect of Particle Size of Graphene, Graphene Oxide and Graphite on Thermal Parameters. C.

[54] Ojha K., Kumar B., Ganguli A.K. (2017). Biomass derived graphene-like activated and non-activated porous carbon for advanced supercapacitors. J. Chem. Sci..

[55] Park J., Kwac L.K., Kim H.G., Park K.-Y., Koo K.W., Ryu D.-H., Shin H.K. (2022). Electromagnetic-Interference-Shielding Effectiveness of Lyocell-Based Carbon Fabrics Carbonized at Various Temperatures. Molecules.

[56] Osman A.I., Farrell C., Al-Muhtaseb A.a.H., Harrison J., Rooney D.W. (2020). The production and application of carbon nanomaterials from high alkali silicate herbaceous biomass. Sci. Rep..

[57] Park J., Kwac L.K., Kim H.G., Shin H.K.J.N. (2021). Fabrication and characterization of waste wood cellulose fiber/graphene nanoplatelet carbon papers for application as electromagnetic interference shielding materials. Nanomaterials.

[58] Aïssa B., Sinopoli A., Ali A., Zakaria Y., Zekri A., Helal M., Nedil M., Rosei F., Mansour S., Mahmoud K.J.C. (2021). Nanoelectromagnetic of a highly conductive 2D transition metal carbide (MXene)/Graphene nanoplatelets composite in the EHF M-band frequency. Carbon.

[59] Hossain M.S., Jahan S.A., Ahmed S. (2023). Crystallographic characterization of bio-waste material originated CaCO_3_, green-synthesized CaO and Ca(OH)_2_. Results Chem..

[60] Sing K.S.W. (1985). Reporting physisorption data for gas/solid systems with special reference to the determination of surface area and porosity (Recommendations 1984). Pure Appl. Chem..

[61] Tompsett G.A., Krogh L., Griffin D.W., Conner W.C. (2005). Hysteresis and Scanning Behavior of Mesoporous Molecular Sieves. Langmuir.

[62] Mohan D., Rajput S., Singh V.K., Steele P.H., Pittman C.U. (2011). Modeling and evaluation of chromium remediation from water using low cost bio-char, a green adsorbent. J. Hazard. Mater..

[63] Fahmi A.H., Samsuri A.W., Jol H., Singh D. (2018). Physical modification of biochar to expose the inner pores and their functional groups to enhance lead adsorption. RSC Adv..

[64] Wang Y., Song T., Zhang P., Huang T., Wang T., Wang T., Zeng H. (2018). Gas-Exfoliation Assisted Fabrication of Porous Graphene Nanosheets Derived from Plumeria rubra for Highly Efficient Photocatalytic Hydrogen Evolution. ACS Sustain. Chem. Eng..

[65] Wang L., Su B., Zhao X., Li X., Han S., Jiang J. (2020). Appropriate Design of Electrode Material by Bio-Derived Nanopores Structure for Symmetric Supercapacitors. Energy Fuels.

[66] Wei F., Bi H., Jiao S., He X. (2020). Interconnected Graphene-like Nanosheets for Supercapacitors. Acta Phys.-Chim. Sin..

[67] Sun Z., Zheng M., Hu H., Dong H., Liang Y., Xiao Y., Lei B., Liu Y. (2018). From biomass wastes to vertically aligned graphene nanosheet arrays: A catalyst-free synthetic strategy towards high-quality graphene for electrochemical energy storage. J. Chem. Eng..

[68] Gharehkhani S., Seyed Shirazi S.F., Pilban Jahromi S., Sookhakian M., Baradaran S., Yarmand H., Ataollahi Oshkour A., Kazi S.N., Basirun W.J. (2015). Spongy nitrogen-doped activated carbonaceous hybrid derived from biomass material/graphene oxide for supercapacitor electrodes. RSC Adv..

[69] Lin S., Ju S., Zhang J., Shi G., He Y., Jiang D. (2019). Ultrathin flexible graphene films with high thermal conductivity and excellent EMI shielding performance using large-sized graphene oxide flakes. RSC Adv..

[70] Cheng Z., Wang R., Wang Y., Cao Y., Shen Y., Huang Y., Chen Y.J.C. (2023). Recent advances in graphene aerogels as absorption-dominated electromagnetic interference shielding materials. Carbon.

[71] Yang Y., Wan C., Huang Q., Hua J. (2022). Pore-Rich Cellulose-Derived Carbon Fiber@Graphene Core-Shell Composites for Electromagnetic Interference Shielding. Nanomaterials.

[72] Zhou X., Wen J., Wang Z., Ma X., Wu H. (2021). Size-controllable porous flower-like NiCo_2_O_4_ fabricated via sodium tartrate assisted hydrothermal synthesis for lightweight electromagnetic absorber. J. Colloid Interface Sci..

[73] Shi S., Jiang Y., Ren H., Deng S., Sun J., Cheng F., Jing J., Chen Y. (2024). 3D-Printed Carbon-Based Conformal Electromagnetic Interference Shielding Module for Integrated Electronics. Nano-Micro Lett..

[74] Li J., Sun H., Yi S.Q., Zou K.-K., Zhang D., Zhong G.-J., Yan D.-X., Li Z.-M. (2023). Flexible Polydimethylsiloxane Composite with Multi-Scale Conductive Network for Ultra-Strong Electromagnetic Interference Protection. Nano-Micro Lett..

