# Supplementary material for: Carbonized Apples and Quinces Stillage for Electromagnetic Shielding
Source: Nanomaterials (Basel). 2024 Nov 23;14(23):1882. doi: 10.3390/nano14231882 (PMC11643710; doi:10.3390/nano14231882)
Supplement: Supplementary file 1 [file nanomaterials-14-01882-s001.zip › nanomaterials-3267413-supplementary.pdf]

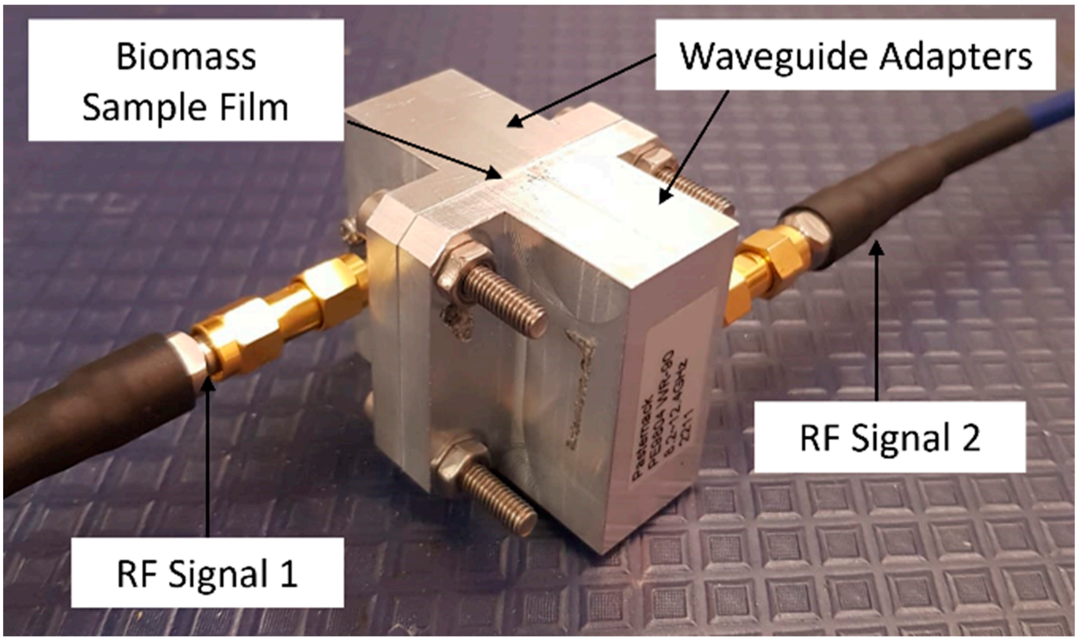

Figure S1. Waveguide-based Measurement Setup for the measurement of shielding of biomass samples.

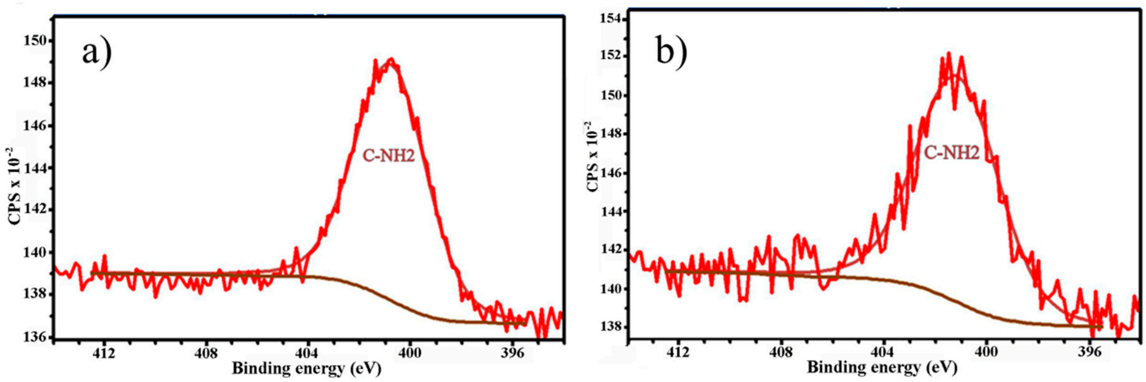

Figure S2. High-resolution spectra of N1s regions for BA (a) and BQ (b) samples.
